# RECON-Dependent Inflammation in Hepatocytes Enhances Listeria monocytogenes Cell-to-Cell Spread

**DOI:** 10.1128/mBio.00526-18

**Published:** 2018-05-15

**Authors:** Adelle P. McFarland, Thomas P. Burke, Alexie A. Carletti, Rochelle C. Glover, Hannah Tabakh, Matthew D. Welch, Joshua J. Woodward

**Affiliations:** a Department of Microbiology, University of Washington, Seattle, Washington, USA; b Molecular and Cellular Biology Program, University of Washington, Seattle, Washington, USA; c Department of Molecular and Cell Biology, University of California, Berkeley, California, USA; University of Illinois at Chicago

**Keywords:** CDNs, *Listeria monocytogenes*, NF-κB, RECON, actin-based motility, cyclic di-AMP, cyclic dinucleotides, iNOS, nitric oxide, oxidoreductase

## Abstract

The oxidoreductase RECON is a high-affinity cytosolic sensor of bacterium-derived cyclic dinucleotides (CDNs). CDN binding inhibits RECON’s enzymatic activity and subsequently promotes inflammation. In this study, we sought to characterize the effects of RECON on the infection cycle of the intracellular bacterium Listeria monocytogenes, which secretes cyclic di-AMP (c-di-AMP) into the cytosol of infected host cells. Here, we report that during infection of RECON-deficient hepatocytes, which exhibit hyperinflammatory responses, L. monocytogenes exhibits significantly enhanced cell-to-cell spread. Enhanced bacterial spread could not be attributed to alterations in PrfA or ActA, two virulence factors critical for intracellular motility and intercellular spread. Detailed microscopic analyses revealed that in the absence of RECON, L. monocytogenes actin tail lengths were significantly longer and there was a larger number of faster-moving bacteria. Complementation experiments demonstrated that the effects of RECON on L. monocytogenes spread and actin tail lengths were linked to its enzymatic activity. RECON enzyme activity suppresses NF-κB activation and is inhibited by c-di-AMP. Consistent with these previous findings, we found that augmented NF-κB activation in the absence of RECON caused enhanced L. monocytogenes cell-to-cell spread and that L. monocytogenes spread correlated with c-di-AMP secretion. Finally, we discovered that, remarkably, increased NF-κB-dependent inducible nitric oxide synthase expression and nitric oxide production were responsible for promoting L. monocytogenes cell-to-cell spread. The work presented here supports a model whereby L. monocytogenes secretion of c-di-AMP inhibits RECON’s enzymatic activity, drives augmented NF-κB activation and nitric oxide production, and ultimately enhances intercellular spread.

## IMPORTANCE

To date, bacterial CDNs in eukaryotes are solely appreciated for their capacity to activate cytosolic sensing pathways in innate immunity. However, it remains unclear whether pathogens that actively secrete CDNs benefit from this process. Here, we provide evidence that secretion of CDNs leads to enhancement of L. monocytogenes cell-to-cell spread. This is a heretofore-unknown role of these molecules and suggests L. monocytogenes may benefit from their secretion in certain contexts. Molecular characterization revealed that, surprisingly, nitric oxide was responsible for the enhanced spread. Pathogens act to prevent nitric oxide production or, like L. monocytogenes, they have evolved to resist its direct antimicrobial effects. This study provides evidence that intracellular bacterial pathogens not only tolerate nitric oxide, which is inevitably encountered during infection, but can also capitalize on the changes this pleiotropic molecule enacts on the host cell.

## INTRODUCTION

A key feature that distinguishes pathogens from innocuous microorganisms is the purposeful breach of the host cell membrane. The cross talk between cytosolic surveillance by innate immune receptors and stress sensors has emerged as a key process by which host cells coordinate and amplify their response to eliminate infectious organisms. Cyclic dinucleotides (CDNs) are central mediators of the host cytosolic immune responses to infection. In addition to the well-characterized STING-dependent inflammatory response, we recently identified the murine aldo-keto reductase (AKR) RECON, encoded by *Akr1c13*, as a cytosolic sensor of bacterial CDNs and a negative regulator of NF-κB-dependent inflammation (1).

Bacterial pathogens have evolved to evade immune surveillance, subvert host signaling downstream of sensing, and resist ensuing antimicrobial responses elicited by the host. Many human pathogens produce cyclic diadenosine monophosphate (c-di-AMP), the only known essential bacterial CDN. In bacteria, c-di-AMP regulates bacterial growth, cell wall homeostasis, and central metabolism (2–4). While the role of c-di-AMP-induced inflammation in the host is best characterized with L. monocytogenes, its effects on other bacteria, including Mycobacterium tuberculosis, Chlamydia trachomatis, Staphylococcus aureus, and group B streptococci (GBS), have recently begun to emerge. GBS evade c-di-AMP-mediated immunity by actively hydrolyzing extracellular nucleotide (5). Similarly, M. tuberculosis hydrolyzes c-di-AMP during infection, and genetic mutants that produce elevated levels of c-di-AMP are highly attenuated (6, 7).

Unlike GBS and M. tuberculosis, which appear to evade this branch of immunity, L. monocytogenes actively secretes c-di-AMP into the host cytosol via the action of several multidrug-resistant (MDR) transporters with relatively minimal effects on pathogenesis *in vivo* (8–11), suggesting that this pathogen has evolved resistance to the host responses that c-di-AMP elicits. In line with this reasoning, we previously reported that augmented inflammation in RECON-deficient hepatocytes restricted growth of *Chlamydia* spp., whereas the replication of L. monocytogenes was unaffected (1). L. monocytogenes has evolved resistance against several key cell-intrinsic host defense mechanisms, including the phagolysosomal pathway, autophagy, and reactive oxygen species (12, 13). However, the antimicrobial effects elicited by RECON, to which L. monocytogenes has seemingly developed resistance, and the consequences on bacterial activity within the host cell are currently unknown.

In this study, we investigated the impact of RECON on the intracellular life cycle of L. monocytogenes growing in hepatocytes. Hepatocytes were studied owing to their high expression of RECON as well as their status as a dominant cellular reservoir of L. monocytogenes during systemic infection (14, 15). Remarkably, we found that L. monocytogenes exhibited enhanced cell-to-cell spread under the hyperinflammatory conditions resulting from the absence of RECON. This phenotype was dependent on NF-κB and ensuing nitric oxide production, the latter of which could enhance L. monocytogenes spread in a variety of host cells. Furthermore, the intracellular secretion of c-di-AMP correlated with L. monocytogenes cell-to-cell spread, a process that was dependent on RECON and NF-κB. Therefore, we propose a model whereby L. monocytogenes secretion of c-di-AMP inhibits RECON’s enzymatic activity, drives augmented NF-κB activation and nitric oxide production, and ultimately enhances intercellular spread.

## RESULTS

### The absence of RECON results in enhanced intercellular spread of L. monocytogenes.

**FIG 1:**
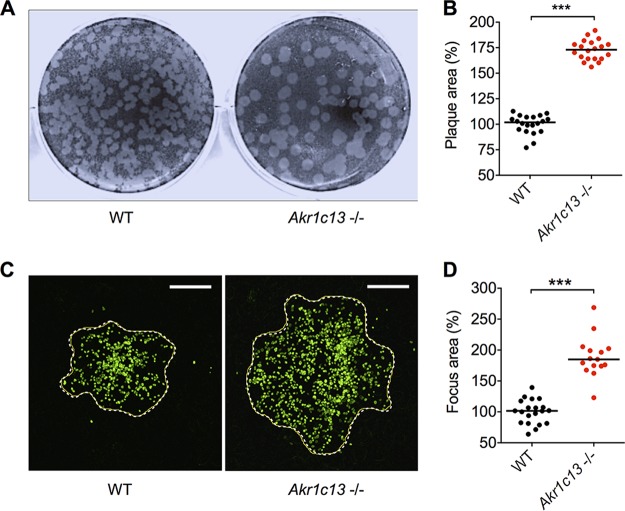
L. monocytogenes enhanced cell-to-cell spread in RECON-deficient hepatocytes. (A) Images of L. monocytogenes plaques (white) spreading through a monolayer of TIB73 cells (black area) for 72 h in a 6-well plate. (B) Quantification of plaque areas shown in panel A. (C) Images of L. monocytogenes cells (green) spreading through TIB73 cells (black area) for 8 h. The dotted line indicates the edge of the focus area. Scale bar, 50 µm. (D) Quantification of focus areas shown in panel C. Median values are indicated by a bar. All data points are shown and are plotted as the percentage of spread in WT TIB73 cells. Data are representative of more than 4 independent experiments. ***, *P* ≤ 0.0001.

As part of its intracellular life cycle, L. monocytogenes utilizes cell-to-cell spread to evade extracellular immune defenses while multiplying within the host. We previously reported that the absence of RECON in the murine embryonic hepatocyte cell line TIB73 did not affect the intracellular replication of L. monocytogenes (1). However, when we examined L. monocytogenes cell-to-cell spread, which can be visualized and quantified based on the presence and size of plaques within a monolayer of cells, we discovered that the loss of RECON resulted in L. monocytogenes plaques that were significantly larger than those seen in wild-type (WT) hepatocytes (Fig. 1A and B). The increased spreading was also observed via microscopy early during infection, where the average area of L. monocytogenes foci in RECON-deficient cells at 8 hours post-infection (hpi) was increased to a similar magnitude and significance as were observed by plaque assay (Fig. 1C and D). We chose to focus on the effects of RECON on L. monocytogenes cell-to-cell spread in hepatocytes versus macrophages, given that hepatocytes are a major cellular reservoir for L. monocytogenes during systemic infection and are the predominant cellular constituent of the liver, where L. monocytogenes focus areas have been visualized *in vivo* (15–20).

**FIG 2:**
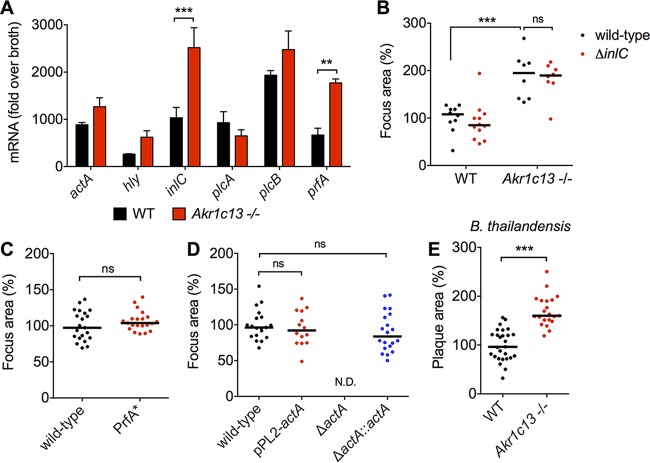
Increased cell-to-cell spread in RECON-deficient cells is likely not due to direct enhancement of L. monocytogenes virulence programs. (A) Expression of the indicated L. monocytogenes virulence genes during infection of WT or *Akr1c13*^−/−^ TIB73 cells at 5 hpi. *16S rRNA* was used an endogenous control. Error bars represent SD of biological duplicates. (B) Quantification of focus areas in WT or Akr1c13^−/−^ TIB73 cells infected with wild-type or Δ*inlC* L. monocytogenes strains. (C and D) Focus areas of the indicated L. monocytogenes strains in WT TIB73 cells. (E) Plaque areas in WT or Akr1c13^−/−^ TIB73 cells infected with B. thailandensis for 16 h. All L. monocytogenes foci were measured at 8 hpi and are plotted as the percentage of the wild-type L. monocytogenes in WT TIB73 cells. Median values are indicated by bars. All foci measured are shown (*n* ≥ 8). Data in panels B to E are representative of at least two independent experiments. **, *P* ≤ 0.001; ***, *P* ≤ 0.0001; ns, not significant.

L. monocytogenes cell-to-cell spread is governed by the coordinated expression of virulence factors involved in vacuolar escape, actin polymerization, and protrusion formation. We measured the expression of several key virulence genes during infection of WT or RECON-deficient hepatocytes to determine if increased virulence gene expression could explain the enhanced spread. We observed increased expression of two virulence genes in RECON-deficient cells, *inlC* and *prfA* (Fig. 2A). *inlC* encodes internalin C, an effector protein involved in promoting protrusion formation by disturbing apical cell tight junctions (21). To test whether the increased expression of *inlC* might explain the enhanced spread, we infected WT or RECON-deficient hepatocytes with a Δ*inlC* strain. We observed that the Δ*inlC* strain had enhanced spread, similar to that of wild-type L. monocytogenes in RECON-deficient cells (Fig. 2B; see also Fig. S1A in the supplemental material). Therefore, we concluded that internalin C was not involved in the enhanced spread.

10.1128/mBio.00526-18.1 FIG S1  Increased cell-to-cell spread in RECON-deficient cells is likely not due to direct enhancement of L. monocytogenes virulence programs. (A) Quantification of plaque areas of wild-type or mutant Δ*inlC* L. monocytogenes cells at 72 hpi in WT or *Akr1c13*^−/−^ TIB73 hepatocytes. (B) Plaque areas of wild-type or PrfA* L. monocytogenes at 72 hpi in WT TIB73 hepatocytes. Data are plotted as the percentage of wild-type L. monocytogenes in WT TIB73 cells, and median values are indicated by the bars. All plaque areas measured are shown. Data are representative of two independent experiments. ns, not significant. Download FIG S1, PDF file, 0.03 MB. Copyright © 2018 McFarland et al. 2018 McFarland et al. This content is distributed under the terms of the Creative Commons Attribution 4.0 International license.

We also observed elevated expression of *prfA* in L. monocytogenes cells infecting RECON-deficient cells (Fig. 2A). PrfA is a master, pleiotropic activator of virulence genes in L. monocytogenes (22). To test if enhanced PrfA activity could promote increased cell-to-cell spread in TIB73 hepatocytes, we infected cells with a strain of L. monocytogenes that expressed a constitutively active form of PrfA (PrfA*) that leads to overexpression of virulence factors (23). The PrfA* strain did not form larger foci or plaques in WT TIB73 cells (Fig. 2C; Fig. S1B), indicating that increased PrfA activity is not sufficient to drive enhanced spread. These data are consistent with previous reports that also found equivalent cell-to-cell spread of wild-type L. monocytogenes and the PrfA* strain (24, 25). Therefore, we concluded that increased PrfA activity did not underlie the enhanced spread in RECON-deficient cells.

Cell-to-cell spread by L. monocytogenes is governed by the polymerization of host cell F-actin into “comet” tails and enables the bacteria to protrude and spread into neighboring cells (26). ActA, which is expressed upon entry into the cytosol, is required for the formation of L. monocytogenes actin tails. Perpetuation of bacterial motility depends on actin accumulation on the bacterial cell surface sufficient to induce and maintain actin nucleation (27). We were not able to directly assess ActA protein levels in TIB73 hepatocytes, because even with high concentrations of bacteria the maximum multiplicity of infection (MOI) was around 0.02. Therefore, we tested whether increased ActA expression in L. monocytogenes could drive enhanced spread in TIB73 hepatocytes by employing a strain that expressed two copies of the *actA* gene. We did not observe increased cell-to-cell spread of this strain in TIB73 cells (Fig. 2D). This was consistent with two previous reports, where a 10 to 20% increase in ActA protein level or 400-fold increase in ActA activity had no effect on L. monocytogenes plaque size (28, 29). Therefore, we concluded that increased levels of ActA were not sufficient to drive enhanced spread and could not account for enhanced L. monocytogenes spread in RECON-deficient cells.

Since the increased L. monocytogenes cell-to-cell spread was not dependent on InlC or PrfA activation, or elevated ActA levels, we reasoned that perhaps the effect on intercellular spread in the absence of RECON may not be L. monocytogenes specific. We infected monolayers of WT or RECON-deficient hepatocytes with the intracellular pathogen Burkholderia thailandensis, which utilizes Arp2/3 complex-mediated cell-to-cell spread analogous to that of L. monocytogenes (30, 31). We found B. thailandensis plaque size was also significantly increased in the absence of RECON (Fig. 2E). Taken together, these results suggested that the increased spread was likely not due to direct enhancement of L. monocytogenes-specific virulence programs but rather may involve alteration of the host cell itself, perhaps though alterations in host factors that affect the Arp2/3 complex and F-actin dynamics.

### L. monocytogenes cells have longer actin tails and increased speed in the absence of RECON.

**FIG 3:**
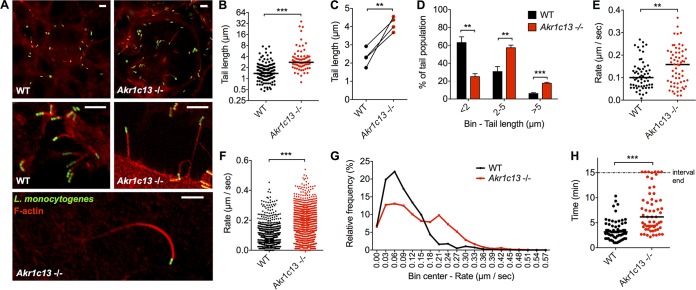
L. monocytogenes actin tail lengths and rates of movement are augmented in RECON-deficient hepatocytes. (A) Images of L. monocytogenes cells (green) associated with F-actin (red) in TIB73 cells at 8 hpi. Scale bars, 5 µm. (B) Quantification of L. monocytogenes actin tail lengths in WT or Akr1c13^−/−^ TIB73 hepatocytes. (C) Data from the same experiment shown in panel B for the mean tail lengths, measured from 4 independent experiments each with 75 to 150 tails quantified. (D) Data from panel C were plotted as binned (<2, 2 to 5, or >5 µm) tail lengths. (E) Average movement rates of L. monocytogenes in TIB73 cells at 6 to 8 hpi. Each data point represents an individual bacterium. Images were captured every 10 s and averaged across at least 6 frames. At least 60 bacteria were measured for each infected cell type. (F and G) Compilation of all L. monocytogenes rates from the individual bacteria measured in panel E. Data are plotted as unbinned (F) or binned (G). (H) Lengths of time individual bacteria (*n* = 60) were associated with their actin tails across 15-min time intervals. (A to H) L. monocytogenes actin tail lengths were measured at 6 to 8 hpi. Median values are indicated by bars. (B, C, and E to G) All data points across multiple experiments are plotted together. **, *P* ≤ 0.001; ***, *P* ≤ 0.0001.

We next examined the effects of RECON deficiency on L. monocytogenes spread by using immunofluorescence microscopy. These studies revealed that L. monocytogenes actin tails were significantly longer in RECON-deficient cells than in WT cells (Fig. 3A and B), a trend that was consistent across multiple experiments (Fig. 3C). The profile of tail lengths shifted from predominantly <2 µm in length in WT cells to between 2 and 5 µm in RECON-deficient cells, with a nearly 3-fold increase in bacteria associated with tails of >5 µm (Fig. 3A and D).

Previous work has demonstrated that L. monocytogenes tail length directly correlates with the speed of individual bacteria (32). However, alterations in the host cell actin disassembly machinery can lead to longer tails without affecting bacterial speed (33, 34). Therefore, we sought to determine whether the longer L. monocytogenes actin tails in RECON-deficient cells correlated with increased rates of movement or whether the longer tails may be the result of decreased actin depolymerization. We tracked and measured the speed of individual bacteria, and when we compared the average rates of movement for each bacterium across the time course, we observed a significant increase in speed in RECON-deficient cells (Fig. 3E). L. monocytogenes is known to exhibit extreme changes in speed and frequent stops (27). Therefore, we also compared the collective rates of movement for each 10-s imaging interval and found that the bacteria in RECON-deficient cells were moving significantly faster between time points than those in WT cells (Fig. 3F). A binned histogram of these data showed a smooth distribution of rates of L. monocytogenes in WT cells, with a peak around 0.05 µm/s (Fig. 3G), which is within the range of L. monocytogenes speeds reported in other cell types (35–39). The histogram for RECON-deficient cells, however, showed a shift toward higher rates, with a second peak emerging at 0.20 µm/s. Therefore, a significant proportion of the intracellular L. monocytogenes population was moving faster than the average rate of movement in the absence of RECON. Remarkably, we also found that in the absence of RECON, L. monocytogenes had significantly increased times of motility and association with their actin tails (Fig. 3H). These data demonstrated that L. monocytogenes exhibits increased rates of movement and longer duration of actin tail association in RECON-deficient cells, suggesting augmented actin-based motility may promote the enhanced cell-to-cell spread.

### Increased NF-κB-dependent inflammation in the absence of RECON’s enzymatic activity drives enhanced L. monocytogenes cell-to-cell spread.

**FIG 4:**
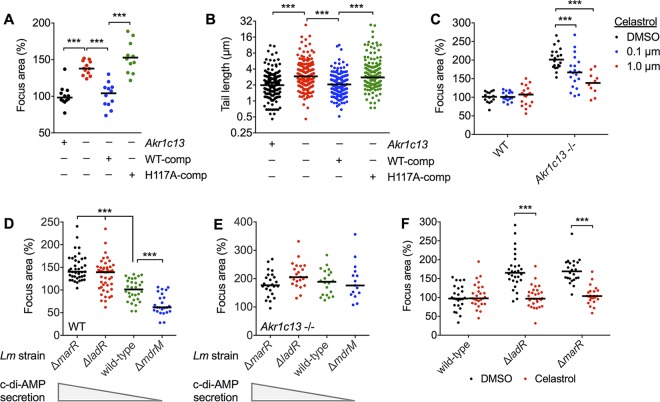
RECON’s enzymatic activity, NF-κB activation, and c-di-AMP secretion control L. monocytogenes intercellular spread. (A and B) L. monocytogenes focus areas (A) or tail lengths (B) in WT, Akr1c13^−/−^, or Akr1c13^−/−^ cells complemented with WT (WT-comp) or H117A (H117A-comp) RECON. (C) Focus areas in WT or Akr1c13^−/−^ TIB73 cells treated with DMSO (control) versus areas for cells treated with the NF-κB inhibitor celastrol (0.1 or 1.0 µM). (D and E) Quantification of focus areas in WT (D) or Akr1c13^−/−^ (E) TIB73 cells infected with the indicated L. monocytogenes strains. Relative levels of c-di-AMP secretion are indicated. (F) Focus areas of wild-type or c-di-AMP hypersecreting strains in WT TIB73 cells treated with DMSO (control) or celastrol (1.0 µM). Median values are indicated by a bar, and all foci measured are shown. Foci and tail lengths were measured at 8 hpi, and foci were plotted as the percentage of wild-type L. monocytogenes in WT TIB73 cells. All data are representative of two independent experiments. ***, *P* ≤ 0.0001.

Investigation of L. monocytogenes virulence gene expression did not yield an explanation for the enhanced spread of these bacteria in RECON-deficient cells (Fig. 2), implicating alterations in the host cell itself as the underlying cause of the phenotype. RECON is an oxidoreductase that belongs to the aldo-keto reductase superfamily of enzymes. We hypothesized that the absence of RECON’s enzymatic activity promoted the enhanced L. monocytogenes spread and increased actin tail lengths. RECON-deficient TIB73 cells were complemented with stably expressed WT RECON or a catalytically dead mutant of RECON (H117A), as previously reported (1). Complementation with WT but not the H117A mutant RECON restored focus areas and L. monocytogenes actin tails to the sizes and lengths, respectively, observed in WT TIB73 cells (Fig. 4A and B), suggesting that loss of RECON’s enzymatic activity drives enhanced cell-to-cell spread of L. monocytogenes.

Our previous characterization of RECON’s enzymatic activity revealed its critical role in controlling NF-κB-dependent inflammation, whereby the absence of RECON activity yielded increased inflammation downstream of Toll-like receptor (TLR) stimulation and L. monocytogenes infection (1). We characterized the effect of the NF-κB inhibitor celastrol on L. monocytogenes cell-to-cell spread and observed a dose-dependent suppression of spread in RECON-deficient hepatocytes (Fig. 4C). Although celastrol has been shown to inhibit mitogen-activated protein kinases as well as NF-κB, our previous work identified RECON as a negative regulator of NF-κB, and therefore the effects of this inhibitor in RECON-deficient hepatocytes is likely via targeting of NF-κB. Overall, these data support the conclusion that the dampening of NF-κB activation by RECON’s enzymatic activity in hepatocytes suppresses L. monocytogenes cell-to-cell spread.

### c-di-AMP secreted by L. monocytogenes promotes intercellular spread in a RECON- and NF-κB-dependent manner.

During infection, c-di-AMP secreted by L. monocytogenes binds to and inhibits the enzymatic activity of RECON (1). Therefore, we tested the hypothesis that L. monocytogenes strains secreting different levels of c-di-AMP might display altered intercellular spread in TIB73 hepatocytes. Multidrug resistance (MDR) transporters are responsible for secretion of c-di-AMP during infection of host cells (8, 9, 40). Importantly, mutants with altered c-di-AMP secretion grow normally in broth and exhibit no morphological defects, unlike mutants with altered c-di-AMP production. We observed that L. monocytogenes strains lacking the major c-di-AMP transporter MdrM (Δ*mdrM*) had reduced cell-to-cell spread in TIB73 hepatocytes but replicated similar to wild-type L. monocytogenes (Fig. 4D; Fig. S2). We also tested L. monocytogenes strains that oversecrete c-di-AMP due to the loss of negative regulators of MdrM (Δ*marR* and Δ*ladR*), and both strains exhibited increased cell-to-cell spread (Fig. 4D).

10.1128/mBio.00526-18.2 FIG S2  L. monocytogenes Δ*mdrM* strain, which secretes lower levels of c-di-AMP and replicates to similar levels as the wild type in hepatocytes. WT TIB73 hepatocytes were infected with L. monocytogenes wild-type or mutant Δ*mdrM* cells and plated for CFU at the indicated time points (*n* = 2). Error bars represent SD. Download FIG S2, PDF file, 0.1 MB. Copyright © 2018 McFarland et al. 2018 McFarland et al. This content is distributed under the terms of the Creative Commons Attribution 4.0 International license.

To determine whether RECON was involved in mediating the differences in cell-to-cell spread of the L. monocytogenes strains secreting different amounts of c-di-AMP, we infected RECON-deficient hepatocytes with these strains. Remarkably, we observed that the focus areas of all the mutant strains, both high and low c-di-AMP secretors, were normalized during infection of the RECON-deficient cells (Fig. 4E). The normalization of responses of mutant Δ*mdrM* cells to that of wild-type L. monocytogenes cells in RECON-deficient hepatocytes suggested that the reduced spread in WT hepatocytes was the result of diminished interaction of c-di-AMP with RECON. Given that the enhanced spreading of wild-type L. monocytogenes in RECON-deficient cells was due to augmented NF-κB activation, we tested whether the increased spread of the c-di-AMP-oversecreting strains was dependent on NF-κB. Treatment with an NF-κB inhibitor blocked the enhanced spread of both Δ*marR* and Δ*ladR* strains (Fig. 4F). Taken together, these data support the conclusion that c-di-AMP inhibition of RECON activity promotes L. monocytogenes cell-to-cell spread via enhanced NF-κB activation.

### Increased nitric oxide production in the absence of RECON enhances L. monocytogenes cell-to-cell spread.

To define the aspect of NF-κB-driven inflammation that increased spread in RECON-deficient cells, we considered previous reports of host cell responses involved in controlling L. monocytogenes spread. Surprisingly, reports of alterations in host cell genotypes that result in increased intercellular spread are rare. Similarly, we found only one report of L. monocytogenes mutants that exhibited significantly enhanced cell-to-cell spread compared to wild-type L. monocytogenes (29); this highlights the significance of the finding that c-di-AMP secretion enhances spread (Fig. 4D). One previous study by Cole and colleagues found that TLR stimulation following L. monocytogenes infection of primary macrophages resulted in elevated nitric oxide production that enhanced cell-to-cell spread (41). The authors also reported that inhibition of inducible nitric oxide synthase (iNOS) during *in vivo* L. monocytogenes infection significantly reduced liver burdens in an ActA-dependent manner. This was the second study to find that nitric oxide could enhance L. monocytogenes burdens in the liver, a phenomenon first reported in 1993 (42).

**FIG 5:**
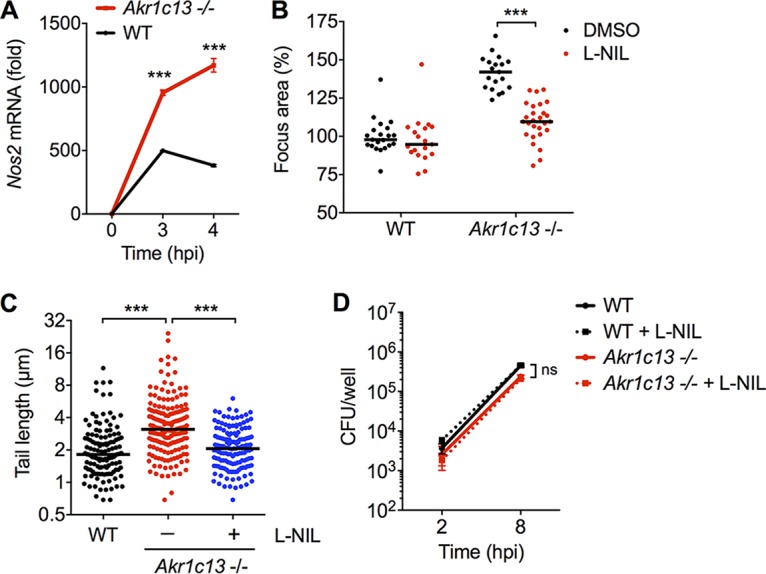
Nitric oxide enhances L. monocytogenes cell-to-cell spread in RECON-deficient hepatocytes. (A) Expression of *Nos2* in WT or Akr1c13^−/−^ TIB73 hepatocytes 2 hpi with L. monocytogenes. *Hprt* served as an endogenous control gene. Error bars represent SEM of technical triplicates. (B and C) L. monocytogenes focus areas (B) and tail lengths (C) in WT or Akr1c13^−/−^ TIB73 cells treated with DMSO (control) versus the iNOS inhibitor l-NIL. (D) WT or Akr1c13^−/−^ TIB73 hepatocytes were infected with L. monocytogenes in the presence of l-NIL or DMSO (control), and CFU were enumerated at the indicated time points (*n* = 3). Error bars represent SD. In panels B and C, median values are indicated by bars. All foci and tails measured at 8 hpi are shown. Foci were plotted as the percentage of wild-type L. monocytogenes in untreated WT cells. All data are representative of at least two independent experiments. ***, *P* ≤ 0.0001; ns, not significant.

These data led us to test the hypothesis that nitric oxide could enhance L. monocytogenes spread in hepatocytes. As we previously reported (1), a striking consequence of augmented NF-κB activity in RECON-deficient hepatocytes is the significantly increased expression of iNOS (Fig. 5A). To test whether the overexpression of iNOS was involved in promoting L. monocytogenes cell-to-cell spread, we treated cells with a specific inhibitor of iNOS, l-N^6^−(1-iminoethyl)-l-lysine (l-NIL). Remarkably, l-NIL reduced the enlarged L. monocytogenes focus areas and longer actin tails in RECON-deficient hepatocytes back to the size and length of those observed in WT hepatocytes (Fig. 5B and C). We did not observe any difference in intracellular bacterial growth in the presence of l-NIL, indicating that the inhibitor was affecting cell-to-cell spread and not L. monocytogenes growth (Fig. 5D).

**FIG 6:**
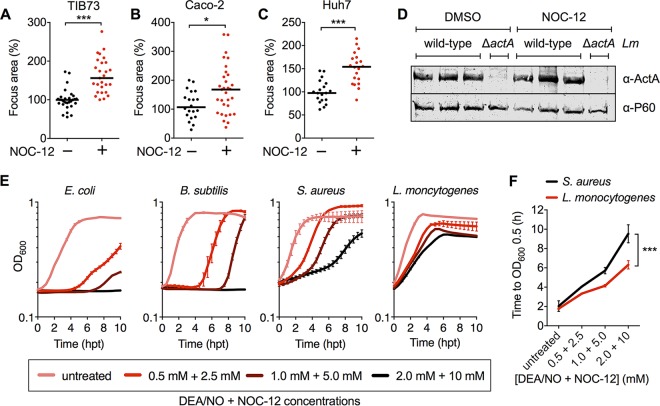
L. monocytogenes cell-to-cell spread is generally enhanced by nitric oxide in nonphagocytic cells. (A to C) Focus areas of L. monocytogenes in WT TIB73 (A), Caco-2 (B), or Huh7 (C) cells treated with the nitric oxide donor NOC-12. (D) Western blot of L. monocytogenes ActA and P60 (loading control) proteins expressed at 5 hpi in Huh7 cells plus or minus treatment with NOC-12, performed in biological triplicates. (E) Growth (OD_600_ values) of Escherichia coli, Bacillus subtilis, Staphylococcus aureus, or Listeria monocytogenes in TSB containing the indicated concentrations of the nitric oxide donors DEA/NO and NOC-12. *n* = 3. Error bars represent SD. (F) Results of the same experiment shown in panel D, with growth times plotted (hours) to reach the indicated OD_600_ in the presence of increasing concentrations of DEA/NO and NOC-12. In panels A to C, median values are indicated by bars. All foci measured at 8 hpi are shown and were plotted as the percentage of wild-type L. monocytogenes in untreated WT cells. All data are representative of two independent experiments, except in panel D, where three biological replicates are shown together. *, *P* ≤ 0.05; ***, *P* ≤ 0.0001.

We also tested whether nitric oxide alone was sufficient to promote L. monocytogenes spread in hepatocytes or whether another aspect of RECON deficiency was also required. Treatment of WT TIB73 hepatocytes with the nitric oxide donor NOC-12 significantly increased L. monocytogenes spread to a level similar to that in RECON-deficient cells (Fig. 6A). To test whether the effect of NOC-12 on L. monocytogenes spread was specific to a unique aspect of murine TIB73 hepatocytes, we also measured L. monocytogenes spread in human Caco-2 cells (enterocyte line) and Huh7 hepatocytes (hepatocellular carcinoma line) (Fig. 6B and C). In both cases, the addition of NOC-12 significantly enhanced L. monocytogenes cell-to-cell spread, indicating that the effect of nitric oxide on L. monocytogenes is not strictly cell type dependent. Earlier results on the effects of RECON deficiency led us to conclude that the virulence protein ActA was not involved in the enhanced L. monocytogenes spreading phenotype (Fig. 2). Unlike TIB73 hepatocytes, Huh7 cells are highly infectible and therefore allowed us to directly assess whether nitric oxide affected ActA protein levels. Robust and similar ActA expression was observed in Huh7 cells with or without NOC-12 treatment, indicating that nitric oxide likely does not enhance spread via increasing ActA protein levels (Fig. 6D). Taken together, these data indicate that RECON’s enzymatic control of nitric oxide production via NF-κB influences L. monocytogenes cell-to-cell spread in hepatocytes.

**FIG 7:**
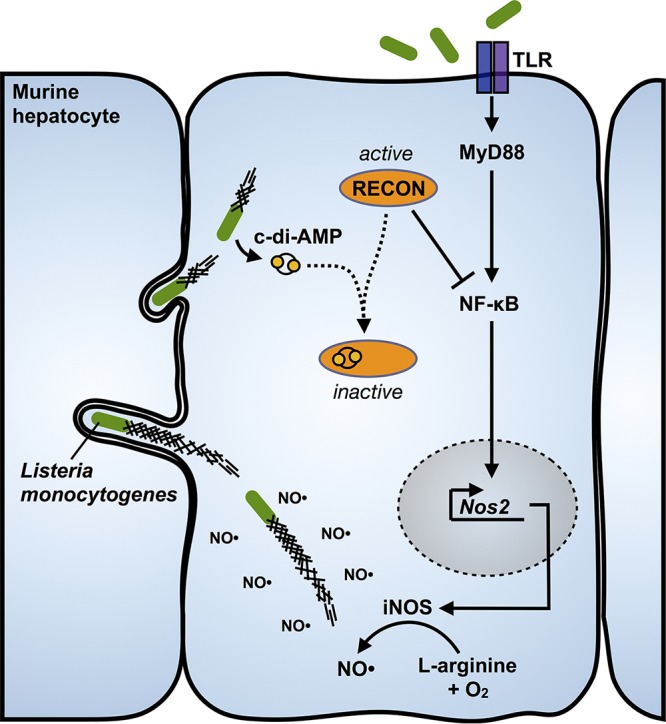
Inhibition of RECON by c-di-AMP promotes L. monocytogenes intercellular spread via increased nitric oxide. During infection, L. monocytogenes secretes c-di-AMP into the cytosol, which is then bound by RECON. Inhibition of RECON’s enzymatic activity by c-di-AMP releases a brake on NF-κB activation downstream of TLR engagement. The augmentation of NF-κB activation leads to increased iNOS levels and nitric oxide production. Elevated nitric oxide promotes elongation of L. monocytogenes actin tails, increased bacterial speed in the cytosol, and enhanced cell-to-cell spread.

It is little appreciated how incredibly resistant L. monocytogenes is to the antimicrobial action of nitric oxide. Many studies have found that L. monocytogenes replication is unaltered in iNOS-deficient macrophages or in the presence of nitric oxide *in vitro*, indicating that nitric oxide itself does not directly contribute to restriction of L. monocytogenes growth (41–45). To demonstrate just how resistant L. monocytogenes is to nitric oxide, we compared the growth of several bacterial species in broth cultures containing supraphysiological concentrations of the nitric oxide donors DEA/NO and NOC-12 (Fig. 6E and F). Staphylococcus aureus colonizes the nasal passage of humans, a site with one of the highest levels of nitric oxide exposure within the host and is considered one of the most nitric oxide-resistant organisms. We found that L. monocytogenes exhibited higher levels of resistance to nitric oxide growth restriction than even S. auerus, while the growth of Escherichia coli and Bacillus subtilis was severely growth impaired by nitric oxide (Fig. 6E and F). Collectively, these studies demonstrated that L. monocytogenes not only replicates unhindered in the presence of nitric oxide but that this antimicrobial host metabolite promotes bacterial spread within host cells (Fig. 7).

## DISCUSSION

Successful colonization and replication within host tissues are predicated upon a pathogen’s capacity to overcome host immunity. Current evidence suggests that several pathogens utilize evasion strategies to limit c-di-AMP-induced inflammation during infection. In contrast, it has been established that L. monocytogenes actively secretes c-di-AMP during infection through the action of a variety of multidrug resistance transporters. These observations suggest that rather than evade host immune sensing of this microbe-associated molecular pattern, L. monocytogenes may utilize other mechanisms to counteract c-di-AMP-mediated immunity to promote infection. Here, we provide evidence that c-di-AMP sensing results in elevated inflammatory responses that are unable to limit bacterial replication in hepatocytes. Additionally, elevated production of host nitric oxide can promote spread of this pathogen. These observations support a model in which L. monocytogenes utilizes c-di-AMP to promote host cell inflammation and, in combination with its evolved resistance to the direct antimicrobial action of nitric oxide, promote a critical aspect of its infection cycle.

While tissue culture models of infection provide a controlled environment in which to interrogate this aspect of L. monocytogenes biology, the complexity of these processes have hampered a clear indication of their *in vivo* impact. Hypersecretion of c-di-AMP is associated with a ~1.5-log reduction of CFU specifically in the liver and not the spleen (8, 11), while reduced c-di-AMP secretion is associated with an ~1-log reduction of CFU in both the liver and spleen (9, 10). Although both hypersecretion and reduced secretion of c-di-AMP leads to a reduction in L. monocytogenes burdens *in vivo*, it is not clear how altered c-di-AMP secretion impacts the host immune response; increased c-di-AMP secretion may increase spread but may also lead to increased immune responses beneficial for bacterial clearance. Additionally, multiple MDRs are known to transport c-di-AMP (8, 9), but their broader roles in L. monocytogenes survival within the host are not fully known. MdrT not only transports c-di-AMP but also is important for resistance to bile acid (10); therefore, *in vivo* data with these mutants are confounded by the probable multiple roles of MDRs in mediating L. monocytogenes survival within host niches.

The effects of nitric oxide on *in vivo* infection are equally complex. Results from this study and those reported previously consistently demonstrate that L. monocytogenes is resistant to the direct antibacterial action of nitric oxide (41–45). Nevertheless, nitric oxide exerts numerous indirect (i.e., nonbacteriostatic/bactericidal) antimicrobial activities, including upregulation of autophagy, pathogen iron deprivation, and inhibition of bacterial secretion systems (46). Additionally, the immunoprotective function of nitric oxide also transcends the intracellular space, owing to its diverse roles in the differentiation of myeloid cells, tissue regeneration, and regulation of T and B cell responses (46). The survival of iNOS-deficient mice administered a sublethal dose of L. monocytogenes is impacted late during infection after day 5, and L. monocytogenes burdens in the liver and spleen are increased by ~2 logs (44, 47). These data suggest that the protection imparted by nitric oxide primarily plays out during the adaptive immune response to L. monocytogenes infection, as T cell responses emerge at day 5 and are ultimately responsible for the clearance of L. monocytogenes in mice (48). Two studies reported the use of chemical inhibitors of iNOS, rather than fully iNOS-deficient mice (41, 42). Interestingly, iNOS inhibition during L. monocytogenes infection reduced burdens primarily in the liver and not the spleen, and this reduction did not occur with infections of L. monocytogenes Δ*actA* mutants (41). Considering that hepatocytes are the dominant cellular reservoir for L. monocytogenes in the liver (14, 15), these data suggest that hepatocytes may be the primary cell type in which nitric oxide promotes spread *in vivo*. Therefore, although nitric oxide production within the context of the entire immune system may act to reduce L. monocytogenes survival, it appears that it also promotes cell-to-cell spread and survival of L. monocytogenes in the liver.

A single study reported a connection between nitric oxide and L. monocytogenes cell-to-cell spread through delayed phagolysosomal maturation in secondarily infected/recipient macrophages (41). In contrast, our investigations in hepatocytes revealed that the absence of RECON did not impact L. monocytogenes cytosolic entry but rather increased actin tails and rates of motility in the cytosol, consistent with distinct mechanisms of nitric oxide-induced spread in hepatocytes versus macrophages. It is not immediately clear how nitric oxide promotes elongated L. monocytogenes actin tails. Nitric oxide can act as a signaling molecule by modulating the activity of target proteins through a variety of mechanisms, including changes in cGMP signaling through activation of soluble guanylate cyclase, direct nitrosylation of cysteines in proteins, and protein nitrosation through the action of nitric oxide-derived peroxynitrite. As such, there may exist several mechanisms by which nitric oxide can modify host proteins and influence L. monocytogenes spread. Previous studies have found that the rate at which L. monocytogenes is moving when it penetrates the plasma membrane to form intercellular protrusions does not positively correlate with speed (33, 35, 49). Thus, it is unclear if the increased rate of L. monocytogenes movement in RECON-deficient cells is directly responsible for increased spread. However, protrusion formation requires at least a minimum rate of movement, and so it is possible that in the absence of RECON, more bacteria meet this threshold requirement to promote protrusion formation and cell-to-cell spread. In line with this, we found that in the absence of RECON, L. monocytogenes cells associate with their actin tails for an increased length of time, which likely provides those bacteria with increased opportunities for protrusion formation. However, future studies are warranted to determine the exact connection between nitric oxide, actin tail lengths, speed, and L. monocytogenes spread.

A broader question that arises from this study is whether enhanced intercellular spread significantly promotes L. monocytogenes virulence. Evidence in support of this proposition is provided by studies of natural isolates of L. monocytogenes. Human clinical isolates, including epidemic strains, are more likely to exhibit enhanced spread compared to the lab reference strain 10403S and to strains from ruminants or food sources (50, 51). The plaque sizes of these hyperspreading isolates are larger by 1.2- to 1.5-fold, which contextualizes the 1.5- to 2-fold increase in spread areas observed in RECON-deficient cells. Interestingly, among natural isolates, cell-to-cell spread phenotypes are not correlated with invasion or replication (e.g., it does not follow that strains that spread more than 10403S also invade better or replicate faster) (50, 52). However, for epidemic strains of L. monocytogenes, enhanced cell-to-cell spread is the most consistent virulence attribute.

We found that hepatocytes treated with nitric oxide showed enhanced L. monocytogenes spread with no impact on L. monocytogenes replication. These data suggest that enhancement of intercellular spread does not directly impact replication from either a nutritional or space perspective. How then might enhanced intercellular spread benefit L. monocytogenes? One of the most frequently ascribed metrics of infection outcome are the bacterial burdens observed during acute infection. However, several other aspects of the infection process are required for a productive infection cycle, including the initial invasion of deeper tissues and transmission from the host. We found that nitric oxide enhanced spread in enterocytes, which may facilitate initial invasion of the intestinal epithelium. Additionally, when L. monocytogenes transits from the bloodstream to the liver, it rapidly infects and resides predominantly within hepatocytes (15). From the liver, L. monocytogenes has been reported to colonize and replicate within the gallbladder following systemic infection (53), which may facilitate transmission through fecal shedding, analogous to Salmonella enterica serotype Typhi (54). Because c-di-AMP and RECON-dependent nitric oxide production affect L. monocytogenes spread within hepatocytes, which are intimately entwined with the gallbladder, it is tempting to speculate that enhanced spread within the liver may expedite entry into the gallbladder to facilitate transmission. Future investigations that interrogate many aspects of the intricate life cycle of this pathogen beyond systemic bacterial burdens will be necessary to fully understand the *in vivo* consequences of alterations in intercellular spread.

In summary, this work establishes that c-di-AMP can promote L. monocytogenes cell-to-cell spread in hepatocytes via engagement of RECON and promotion of nitric oxide production downstream of NF-κB activation. These findings provide the basis for novel avenues of inquiry, such as the impact that c-di-AMP secretion and nitric oxide have on *in vivo* intercellular spread and host-to-host transmission of L. monocytogenes.

## MATERIALS AND METHODS

### Bacterial strains and culture conditions.

Listeria monocytogenes 10403S strains were streaked onto brain heart infusion (BHI) agar, grown overnight at 37°C, and then stored at 4°C for up to 1 month. For infections, one colony of L. monocytogenes was inoculated into 3 ml of BHI and grown overnight, static, at 30°C. The next morning, 1 ml of culture (normalized to an optical density at 600 nm [OD_600_] of 1.1) was centrifuged and washed 3 times with 1 ml of sterile 1× phosphate-buffered saline (PBS). The pellet was resuspended in 1 ml of 1× PBS for infections. Burkholderia thailandensis strain E264 Δ*motA2* (55) was streaked onto BHI agar, grown overnight at 37°C, and stored at room temperature for up to 1 week. For infections, one colony of B. thailandensis was inoculated into 2 ml of BHI and grown overnight with shaking at 37°C. The next day, the bacteria were diluted 40× into 2 ml of fresh BHI and grown with shaking at 37°C for 4 h until an OD_600_ of 3 to 4 was reached. The bacteria were then centrifuged and washed 2 times with 1 ml of sterile 1× PBS, and the pellet was resuspended in 1 ml of 1× PBS for infections.

### Cell lines.

TIB73 is a spontaneously immortalized hepatocyte cell line from a normal BALB/c embryo liver (sex unknown, as it was not provided by the original depositor). The cell line was authenticated by and purchased from ATCC. TIB73 hepatocytes deficient in RECON (*Akr1c13*^−/−^) generated by CRISPR/Cas9-mediated mutagenesis have been described previously (1). Huh7 and Caco-2 are cancer cell lines derived from human males with hepatocellular carcinoma and colon adenocarcinoma, respectively. Huh7 and Caco-2 cells were obtained from Ram Savan (University of Washington) (56, 57) and tested negative for mycoplasma contamination. They were authenticated by their morphology, infectibility, and response to stimuli. Cell lines were grown at 37°C in 5% CO_2_ in phenol red-free Dulbecco’s modified Eagle’s medium (DMEM) with 10% heat-inactivated fetal bovine serum (FBS; 20% for Caco-2 cells) and supplemented with 2 mM sodium pyruvate and 1 mM l-glutamine. For passaging, cells were maintained in Pen-Strep (100 U/ml) but were plated in antibiotic-free media for infections.

### Bacterial infections of cell lines.

A total of 1 × 10^6^/3 ml (TIB73, Huh7) or 1.75 × 10^6^/3 ml (Caco-2) cells were seeded per well in 6-well plates the day before infection. For foci analysis, cells were plated on top of collagen-coated coverslips. On the morning of infection, the cells were washed 2 times in 1× PBS just prior to infection. L. monocytogenes strains were diluted 1:100 (for growth curves, foci, actin tail length, and RNA analyses), 1:500 (for TIB73 plaque assays), or 1:15,000 (for Huh7, Caco-2 foci analyses) in prewarmed cell culture medium containing 0.1% FBS. Two milliliters of diluted bacteria was overlaid onto the host cells and placed at 37°C for 1 h. Following infection, the cells were washed 2 times with 1× PBS and placed into complete medium containing gentamicin (50 µg/ml) to kill extracellular bacteria. For TIB73 growth curves, cells were washed 2 times with 1× PBS, lysed in 500 µl cold 1× PBS with 0.1% Triton X-100, and plated as previously described (58). TIB73 plaque assays with L. monocytogenes were conducted as previously described (59).

For live cell imaging of L. monocytogenes in WT and RECON-deficient TIB73s, cells were plated at 5 × 10^5^ per dish onto 20 mM MatTek dishes 24 h prior to infection in FluoroBrite DMEM containing 10% FBS. Overnight cultures of L. monocytogenes expressing green fluorescent protein (GFP) under the *actA* promoter (pPL2-actA-GFP) were washed 2 times in 1× PBS and diluted 1:100 into the culture dish. Gentamicin (50 µg/ml) was added at 1 hpi. For B. thailandensis infections of TIB73 cells, the washed bacteria were diluted 1:4,000, 1:20,000, or 1:100,000 in 2 ml of cell culture medium, and each dilution was added to a well of TIB73 cells in a 6-well format. Cells were then incubated at 37°C for 30 min, washed 2 times with 1× PBS, and overlaid with medium containing 5% FBS, 0.7% agarose, and 1 mg/ml kanamycin. Infected cells were incubated at 37°C, and plaques were imaged at 16 hpi.

### Microscopy.

For microscopic analysis of L. monocytogenes foci and actin tails, cells were infected for 8 h, washed 2 times with 1× PBS, and fixed in 3.5% formaldehyde for 15 min at room temperature. The coverslips with attached cells were washed in Tris-buffered saline (TBS) with 0.1% Triton X-100 and blocked in TBS with 1% bovine serum albumin (BSA). Cells were stained in TBS with 1% BSA with rabbit L. monocytogenes O antiserum (1:100 dilution), washed, incubated with a goat anti-rabbit IgG secondary Alexa Fluor 488 conjugate (1:200 dilution) and Alexa Fluor 568 phalloidin (F-actin probe; 1:1,000 dilution), and washed again. The coverslips were mounted and imaged with a Keyence BZ-X710 microscope. Quantitative analyses of focus areas and tail lengths were performed with ImageJ software. B. thailandensis plaques were imaged on a Leica DM IL LED microscope with a 10× objective. Images were analyzed for focus size using ImageJ.

### L. monocytogenes rate analysis.

WT or *Akr1c13*^−/−^ TIB73 hepatocytes were transduced with rLV-Ubi-LifeAct lentivirus (ibidi) and selected according to the manufacturer’s instructions to generate red fluorescent protein (RFP)-tagged F-actin for live cell imaging. To measure L. monocytogenes movement rates, these cells were infected for 6 to 8 h prior to imaging. Images were captured using a Nikon Ti Eclipse microscope with a Yokogawa CSU-XI spinning disc confocal, a Clara Interline charged-couple-device camera, 60× (1.4 numerical aperture) Plan Apo objective, and MetaMorph software. Two *Z*-stacks were captured at 10-s intervals for 15 min per field of view. Individual movements were then tracked using ImageJ software with the Manual Tracking plug-in. A minimum of 60 individual bacteria were tracked for each infected cell type. Bacteria that did not migrate ~20 µm from their original location were not tracked, and tracking was stopped when the bacteria stopped migrating.

### Isolation of L. monocytogenes RNA from host cells.

Six 60- by 30-mm dishes of TIB73 cells were infected for 5 h. At the time of harvest, cells were washed 2 times with 1× PBS and then lysed in the dishes on ice for 5 min with 3 ml of 0.1% SDS, 1% acidic phenol, and 19% ethanol (EtOH) to release residing bacteria. Lysates were pooled, and the released bacteria were pelleted at 4,500 rpm for 10 min at 4°C. Pellets containing bacteria were resuspended in 500 µl of 1× PBS, diluted with 500 µl cold methanol, and stored at −20°C until RNA extraction. At the time of extraction, bacteria were pelleted at 4,500 rpm for 10 min at 4°C. Pellets were resuspended in 400 µl of diethyl pyrocarbonate-treated water with 50 mM NaO-acetate (NaOAc; pH 5.2) and 10 mM EDTA. This bacteria-containing buffer was mixed with 80 µl of 10% SDS and 400 µl 1:1 acidified phenol-chloroform, vortexed for 10 min, and then incubated at 65°C for 10 min. The contents were then poured into phase lock tubes and centrifuged for at 17,000 rpm for 5 min. Four hundred microliters of the aqueous layer was transferred to 40 µl 3 M NaOAc (pH 5.2) plus 1.0 ml 100% ethanol, vortexed, placed at −20°C for 1 h, and centrifuged at 17,000 rpm for 30 min at 4°C. Samples were aspirated, washed with 500 µl of 70% EtOH, and centrifuged at 17,000 rpm for 10 min at room temperature. Samples were aspirated and dried in a SpeedVac for 2 min, and RNA pellets were resuspended in 50 µl of RNase-free water.

### qRT-PCR.

For quantitative reverse transcription-PCR (qRT-PCR), RNA was extracted, DNase treated, and reverse transcribed and assayed for gene expression by using SYBR green (L. monocytogenes genes) or TaqMan (host cell genes) chemistries according to the manufacturers’ instructions, as previously described (1).

### Chemical inhibitors.

The NF-κB inhibitor celastrol (used at 0.1 and 1 µM as indicated in the figures and their legends) was added to cells for 1 h prior to infection. Cells were maintained in the presence of celastrol during infection and then again after bacteria were washed off, out to 8 hpi. All inhibitors were reconstituted in dimethyl sulfoxide (DMSO), which by itself was used as a control treatment. For the cell culture experiment, l-NIL and the nitric oxide donor NOC-12 were added to a final concentration of 1 mM and 50 µM, respectively, immediately following a 1-h infection of the cells with L. monocytogenes. For *in vitro* bacterial growth curves, bacteria were grown overnight in tryptic soy broth (TSB) and subcultured 1:50 in fresh TSB in 96-well plates (200 µl, final volume). The nitric oxide donors DEA/NO and NOC-12 were added (concentrations areindicated in figures and/or legends), and bacteria were grown at 37°C with shaking in a BioTek Synergy plate reader.

### Immunoblotting.

For immunoblotting, 0.7 × 10^6^ Huh7 cells were plated in 12-well plates and rested overnight. The cells were infected for 1 h with L. monocytogenes at a 1:1,000 dilution of bacteria of overnight culture as described above (see “Bacterial infections of cell lines”). NOC-12 (50 µM) was added to the cells 1 hpi, and at 5 hpi the cells were lysed in Laemmli sample buffer with 5% 2-mercaptoethanol as previously described (28). Western analyses were run using nitrocellulose membranes blocked in 5% nonfat dry milk in 1× TBS for 45 min at room temperature, followed by overnight incubation in primary antibodies diluted 1:5,000 in 5% BSA in 1× TBS with Tween. Antibody information is provided in the Table S1.

10.1128/mBio.00526-18.3 TABLE S1  Key resources for information on antibodies, bacterial strains, cell lines, chemicals, commercial assays, oligonucleotide sequences, and software used in this study. Download TABLE S1, PDF file, 0.1 MB. Copyright © 2018 McFarland et al. 2018 McFarland et al. This content is distributed under the terms of the Creative Commons Attribution 4.0 International license.

### Quantification and statistical analysis.

Data were analyzed using Prism 6 software. An unpaired, nonparametric (Mann-Whitney) two-tailed *t* test was used to determine significance of all data except for those in Fig. 2A, 5A, and 6E (for which two-way analysis of variance [ANOVA] with Bonferroni’s multiple comparisons test), Fig. 3B, E, F, and H (unpaired, nonparametric, two-tailed Kolmogorov-Smirnov test comparing the cumulative distributions); Fig. 4A, B, D, and E (ordinary one-way ANOVA with Bonferroni’s multiple comparisons test). *P* values correlate with symbols (*, *P* ≤ 0.05; **, *P* ≤ 0.001; ***, *P* ≤ 0.0001) and are also denoted in the figure legends. The values for and identify of *n* (biological replicates) as well as precision measures (e.g., median, standard deviation [SD], and standard error of the mean [SEM]) are indicated in the figure legends. Investigators remained unblinded to sample identities throughout. No data were excluded from the statistical analyses.

### Reagent and resource sharing.

Further information and requests for resources and reagents should be directed to and will be fulfilled by the corresponding author, Joshua J. Woodward.
