# Supplementary material for: RECON-Dependent Inflammation in Hepatocytes Enhances Listeria monocytogenes Cell-to-Cell Spread
Source: mBio. 2018 May 15;9(3):e00526-18. doi: 10.1128/mBio.00526-18 (PMC5954220; doi:10.1128/mBio.00526-18)
Supplement: FIG S1 [file mbo003183888sf1.pdf]

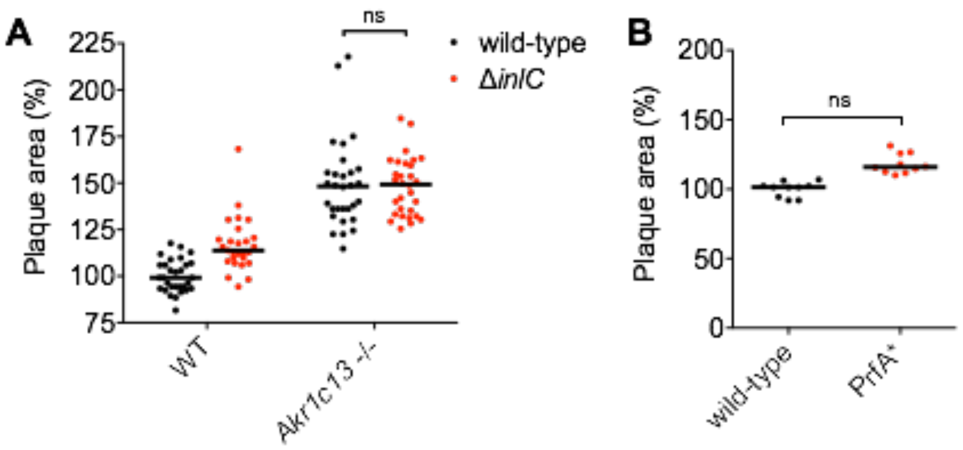

Supplemental Figure 1 (Related to Main Figure 2). Increased cell-to-cell spread in RECON-deficient cells is likely not due to direct enhancement of *L. monocytogenes* virulence programs.

(A) Quantification of plaque areas of wild-type or Δ*inlC L. monocytogenes* at 72 hpi in WT or *Akr1c13*-/- TIB73 hepatocytes.

(B) Plaque areas of wild-type or PrfA* *L. monocytogenes* at 72 hpi in WT TIB73 hepatocytes.

Data are plotted as percent of wild-type *L. monocytogenes* in WT TIB73 cells, and median values are indicated by a bar. All plaque areas measured are shown. Data are representative of two independent experiments. ns = not significant.
