# Supplementary material for: RECON-Dependent Inflammation in Hepatocytes Enhances Listeria monocytogenes Cell-to-Cell Spread
Source: mBio. 2018 May 15;9(3):e00526-18. doi: 10.1128/mBio.00526-18 (PMC5954220; doi:10.1128/mBio.00526-18)
Supplement: FIG S2 [file mbo003183888sf2.pdf]

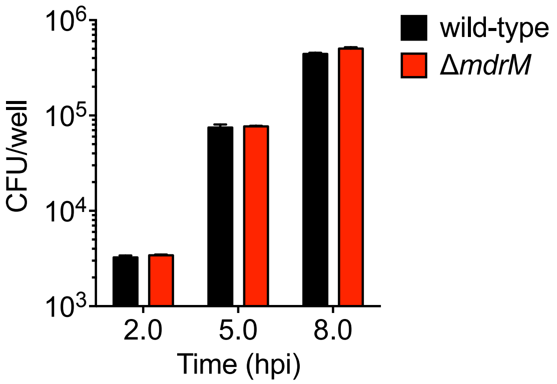

Supplemental Figure 2 (Related to Main Figure 4). ***L. monocytogenes* Δ*mdrM*, which secretes lower c-di-AMP, replicates to similar levels as wild-type in hepatocytes.** WT TIB73 hepatocytes were infected with *L. monocytogenes* wild-type or Δ*mdrM* and plated for CFU at the indicated time points (N = 2). Error bars represent ± SD.
