# Supplementary material for: RECON-Dependent Inflammation in Hepatocytes Enhances Listeria monocytogenes Cell-to-Cell Spread
Source: mBio. 2018 May 15;9(3):e00526-18. doi: 10.1128/mBio.00526-18 (PMC5954220; doi:10.1128/mBio.00526-18)
Supplement: TABLE S1 [file mbo003183888st1.pdf]

Key resources table

REAGENT or RESOURCE	SOURCE	IDENTIFIER
Antibodies		
Difco Listeria O Antiserum Poly Types 1 & 4	Becton Dickinson	Cat#223021
Alexa Fluor 568 Phalloidin	Thermo Fisher Scientific	Cat#A12380
Anti-ActA polyclonal antibody raised against the mature N terminus of the ActA protein	Michelle Reniere	Reniere *et al.* (Nature, 2015); Lauer *et al.* (I&I 2008)
Anti-*Listeria* spp. P60 mAb (P6017)	Adiopgen	Cat#AG-20A-0023
IRDye 680RD Goat anti-Mouse IgG (1:10,000 dilution)	Licor	Cat#926-68070
IRDye 800CW Goat anti-Rabbit IgG (1:10,000 dilution)	Licor	Cat#926-32211
Bacterial and Virus Strains		
rLVUbi-LifeAct-TagRFP: lentiviral vector	Ibidi	Cat#60142
Listeria monocytogenes 10403S wild-type	Provided by Dan Portnoy	JW06
Listeria monocytogenes 10403S Δ*marR*	Provided by Dan Portnoy; Crimmins et al., 2008	JW524
Listeria monocytogenes 10403S Δ*ladR*	Provided by Dan Portnoy; Crimmins et al., 2008	JW523
Listeria monocytogenes 10403S Δ*mdrM*	Provided by Dan Portnoy; Crimmins et al., 2008	JW07
Listeria monocytogenes 10403S Δ*inlC*	Provided by Dan Portnoy	JW551
Listeria monocytogenes 10403S PrfA*	Provided by Dan Portnoy; Wong and Freitag, 2004	JW130
Listeria monocytogenes 10403S Δ*actA*	Provided by Dan Portnoy	JW367
Listeria monocytogenes 10403S pPL2:*actA*	This study	JW618
Listeria monocytogenes 10403S Δ*actA*::*actA*	This study	JW620
Listeria monocytogenes pPL2-actA-GFP	Provided by JD Sauer	JW480
Burkholderia thailandensis strain E264 Δ*motA2*	French et al., 2011	N/A
Escherichia coli XL1 Blue	N/A	N/A
Bacillus subtilis strain 168	Provided by Houra Merrikh	HM01/JW227
Staphylococcus aureus strain Newman	Provided by Dan Portnoy	N/A
Chemicals, Peptides, and Recombinant Proteins		
DMEM, high glucose, no glutamine, no phenol red	Thermo Fisher Scientific	Cat#31053036
Sodium pyruvate	Thermo Fisher Scientific	Cat#11360070
L-Glutamine	Thermo Fisher Scientific	Cat#25030081
Characterized Fetal Bovine Serum	Thermo Fisher Scientific	Cat#SH30071.03
Penicillin-Streptomycin (10,000 U/mL)	Thermo Fisher Scientific	Cat#15140122
Bovine Serum Albumin (BSA)	Fisher BioReagents	Cat#BP9700100

Trypsin-EDTA (0.05%)	Thermo Fisher Scientific	Cat#25300054
Brain Heart Infusion Broth	Research Products International	Cat#B11000
Tryptic soy broth (TSB)	BD Dianostics	Cat#211825
Gentamicin (10 mg/mL)	Thermo Fisher Scientific	Cat#15710072
Dimethyl sulfoxide	Sigma-Aldrich	Cat#D2650
Celastrol	InvivoGen	Cat#ant-cls
L-NIL hydrochloride	Cayman Chemical	Cat#80310
DEA NONOate (DEA/NO)	Cayman Chemical	Cat#82100
NOC-12	Sigma-Aldrich	Cat#487955
Pierce 16% formaldehyde	Thermo Fisher Scientific	Cat#28908
Neutral Red solution	Sigma-Aldrich	Cat#N6264
BioCoat Collagen I 22mm round coverslips	Corning	Cat#354089
Prolong Diamond Antifade Mountant	Thermo Fisher Scientific	Cat#P36961
Critical Commercial Assays		
TURBO DNA-free Kit	Thermo Fisher Scientific	Cat#AM1907
iScript cDNA synthesis kit	Bio-Rad	Cat#1708891
Maxima SYBR Green/ROX qPCR Master Mix	Thermo Fisher Scientific	Cat#K0221
RNAqueous Total RNA Isolation Kit	Thermo Fisher Scientific	Cat#AM1912
TaqMan pre-designed assay: murine *Nos2*	Thermo Fisher Scientific	Mm00440502_m1
TaqMan pre-designed assay: murine *Hprt*	Thermo Fisher Scientific	Mm03024075_m1
Experimental Models: Cell Lines		
TIB73	ATCC	ATCC Cat# BNL CL.2 TIB-73; RRID:CVCL_4383
TIB73 mutant *Akr1c13*	McFarland et al., 2017	N/A
TIB73 mutant *Akr1c13* with MSCV-*Akr1c13*-WT	McFarland et al., 2017	N/A
TIB73 mutant *Akr1c13* with MSCV-*Akr1c13*-H117A	McFarland et al., 2017	N/A
Huh7	Provided by Ram Savan; McFarland et al., 2014; Jarret et al., 2016	RRID:CVCL_0336
Caco-2	Provided by Ram Savan; Lim et al., 2016	RRID:CVCL_0025
Oligonucleotides		
Lm 16S forward primer: CAAGCGTTGTCCGGATTTATTG	This paper	N/A
Lm 16S reverse primer: GCACTCCAGTCTTCCAGTTT	This paper	N/A
Lm actA forward primer: ACGGGACCAAGATACGAA	This paper	N/A
Lm actA reverse primer: GCATGCTAGAATCTAAGTCAC	This paper	N/A
Lm hly forward primer: CGCGGATGAATTCGATAG	This paper	N/A
Lm hly reverse primer: GTCATACCCGGGAAATCAATG	This paper	N/A
Lm inlC forward primer: AATTCCCACAGGACACAACC	This paper	N/A
Lm inlC reverse primer: CGGGAATGCAATTTTTCACTA	This paper	N/A
Lm plcA forward primer: TTCGGGGAATTCCATGATTAG	This paper	N/A
Lm plcA reverse primer: CACTACTCCCGGGACTGAG	This paper	N/A
Lm plcB forward primer: CCAGTAGGATCCACTGTATC	This paper	N/A

Lm plcB reverse primer: CTTATTTCCCGGGTTTTGCTAATG	This paper	N/A
Lm prfA forward primer: CAGCTGAGCTATGTGCGAT	This paper	N/A
Lm prfA reverse primer: ACCAATGGGATCCACAAG	This paper	N/A
Software and Algorithms		
ImageJ	ImageJ Software	https://imagej.nih.gov/ij/
BZ-X700 series microscope analysis application	Keyence	BZ-H3AE
GraphPad Prism 6	GraphPad Software	http://www.graphpad.com/scientificsoftware/prism/
Metamorph	Molecular Devices	https://www.moleculardevices.com/systems/metamorph-research-imaging
Leica Application Suite, V4	Leica Microsystems	https://www.leica-microsystems.com/products/microscope-software/
